# The Effect of Duck Breeds on Carcass Composition and Meat Quality at Different Slaughter Ages

**DOI:** 10.3390/ani15142106

**Published:** 2025-07-16

**Authors:** Lixia Wang, Xing Chen, Yu Yang, Shengqiang Ye, Ping Gong, Yanan Wang, Mingli Zhai, Yan Wu, Yunguo Qian

**Affiliations:** 1 Institute of Animal Husbandry and Veterinary, Wuhan Academy of Agricultural Sciences, Wuhan 430208, China; wxmmrc2008@163.com (L.W.); ligang1997@126.com (X.C.); yang007yu@outlook.com (Y.Y.); yeshq0805@163.com (S.Y.); gongping102@126.com (P.G.); wangyananhzau@163.com (Y.W.); minglizhai@gmail.com (M.Z.); 2 Institute of Animal Science and Veterinary Medicine, Hubei Academy of Agricultural Sciences, Wuhan 430064, China; wuyanwh@163.com

**Keywords:** duck, slaughter traits, meat quality traits, analysis of variance

## Abstract

Meat quality is influenced by factors such as age, breed, slaughter weight, and nutrition. This study investigated the growth performance, slaughter performance, and meat quality of ducks across different breeds and ages. Results indicated that at the same age, significant differences in body weight were observed among breeds (*p* < 0.05), with the weight ranking in descending order as follows: Cherry Valley ducks (C) > Wuqin 10 meat ducks (W) > Mianyang Partridge ducks (M) > Liancheng White ducks (L). A comparison of the same breed across different ages revealed that the pectoral muscle ratio tended to increase with age, whereas the leg muscle ratio showed the opposite trend; however, total meat production gradually rose. At all three growth stages, C ducks exhibited higher body weight and meat yield than the other breeds. W ducks demonstrated excellent meat quality traits and appropriate meat production, with indices such as shear force, water-holding capacity, and fat content all higher than those of the other breeds. L ducks and M ducks had relatively lower body weight and meat production compared to the other breeds, yet their shear force and water-holding capacity were superior to those of C ducks. The analysis of meat quality at different times showed that across all breeds, shear force, meat color, muscle fiber diameter, crude protein content, and fat content increased with age, while drip loss rate and muscle fiber density decreased. A comprehensive multi-index evaluation model for duck meat quality under different breeds was established, along with a four-factor principal component model (Z1, Z2, Z3, Z4). Using the comprehensive ranking equation K, the meat quality performance of different breeds at various ages, in descending order, was as follows: 63-day-old W > 90-day-old M > 63-day-old C > 90-day-old L > 63-day-old M > 90-day-old C > 63-day-old L > 90-day-old W > 42-day-old C > 42-day-old W. This study not only provides a theoretical basis for evaluating meat quality traits in different duck breeds but also offers insights for breed selection and age-related quality optimization.

## Simple Summary

Understanding the influence of animal breed and age on meat quality is of critical importance, as it will significantly impact marketing strategies. In this study, four major meat duck breeds were comprehensively and systematically examined. A detailed comparison of their meat production performance and meat quality characteristics was conducted, followed by a ranking of the meat quality of these duck breeds at different ages. The results of this research will provide valuable reference points for meat duck processing operations and consumer preferences, thereby offering practical guidance for industry stakeholders to more effectively optimize production and meet market demands.

## 1. Introduction

China is a major producer of meat ducks and recent statistics show that global meat duck production in 2022 reached approximately 5.59 billion birds. Of these, China accounted for 4.002 billion birds (72% of the global quantity) and 69% of global production by weight, ranking first worldwide in both metrics [1]. In 2023, China’s total livestock and poultry meat output reached 105.7 million tons, including 10.23 million tons (9.7% of total meat production) of duck meat. Thus, duck meat is a critical component of China’s meat supply [2].

Consumer preferences in the Chinese market are shifting from quantitative to qualitative demands. Meat quality is a growing concern and consumption patterns demonstrate diverse regional preferences, including Beijing roast duck, soy-sauce braised duck, and salt-cured pressed duck. Three primary meat duck categories have emerged to meet these product-specific requirements: the Peking duck-type breed, small-to-medium-sized premium breeds, and spotted ducks. Cherry Valley ducks are fast-growing with large body sizes, enabling early and high meat production—qualities that make them well-suited for roast duck. Wuqin 10 meat ducks, a medium-growing cultivated breed officially recognized by the National Committee for Livestock and Poultry Genetic Resources, are ideal for sauce duck. Liancheng White ducks and Mianyang Partridge ducks are slow-growing indigenous Chinese egg-type breeds; despite their smaller size, they boast excellent meat flavor, making them suitable for both sauce duck and duck soup.

Meat quality can be influenced by factors such as sex, age, breed, slaughter weight, nutrition, slaughter procedure, aging time, and processing [3,4,5]. The effects of animal breed, age, and weight on meat quality traits are particularly important because they affect marketing decisions [6,7]. Studies have demonstrated that Cherry Valley ducks exhibit superior body weight gain, carcass yield, and protein content compared to Liancheng White ducks. Conversely, Liancheng White ducks surpass Cherry Valley ducks in flavor-related compounds such as IMP, amino acids, and fatty acids. Additionally, the nutritional components and flavor substances in breast muscles increased with age, with growth rates slowing in the later stages [7]. Peking ducks have high lipid deposition capacity and Liancheng White ducks have the high medicinal function [8]. In fast- and slow-growing ducks, the significant clear differences of fiber diameter, fiber density, fiber cross-sectional area, moisture content, release water, and intramuscular fat content were significantly affected by the breeds [9]. The differences in duck genotype had a significant effect on the water, fat content, shear force, etc. in pectoralis major muscle [10]. Another study indicates that age exerted a greater effect on the observed meat quality traits of breast meat than breed [11].

However, the influence of breed and age on meat quality in typical meat duck breeds remains unclear. In this study, four duck breeds—Cherry Valley duck (C), Wuqin 10 meat duck (W), Liancheng White duck (L), and Mianyang Partridge duck (M)—were evaluated at three growth stages (42, 63, and 90 days old) to investigate how different breeds and growth stages affect carcass traits and meat quality. The results of this study provide reference values for meat duck processing and consumer preferences.

## 2. Materials and Methods

### 2.1. Animals, Diets, and Experimental Design

All animal experimental procedures were approved by the Animal Ethics Committee of the Wuhan Academy of Agricultural Sciences (ethical approval number: 2021-620-000-001-02).

A total of 640 1-day-old ducks, comprising 160 Wuqin 10 meat ducks (W), 160 Liancheng White ducks (L), 160 Mianyang Partridge ducks (M), and 160 Cherry Valley ducks (C), with an equal sex distribution (50% male/50% female), were selected for this study. The initial weight of each breed was within the average range. Each breed had 4 replicates, with 40 birds per replicate (50% male/50% female). Individuals wore foot numbers and were housed based on the “duck shed + sports field + play pool” culture pattern. The experiment lasted 90 days. When the ducks were 1 to 3 days old, the temperature within the duck house was maintained at 32 °C, then gradually decreased to a temperature ranging from 10 to 25 °C. The ambient humidity was maintained at 65–75%. Ducks were fed complete formula feed at Gantang Breeding Duck Farm in Huangpi District, Wuhan City, and they had free access to food and water during the entire trial period. Composition and nutrient level of experimental animal diets are presented in Table 1.

### 2.2. Slaughter Surveys and Sample Collection

Ducks were weighed after fasting for 12 h at 42, 63, and 90 days of age. At each stage, four ducks (two males and two females) with similar weights were selected from each replicate group and anesthetized with pentobarbital sodium, after which they were sacrificed via jugular puncture. Slaughter performance was measured and calculated following the standard issued by the “Terminology and measurement calculation method for poultry performance” [12].

### 2.3. Meat Quality

Using the above euthanasia methods, another four ducks (two males and two females) are selected for each replicate group. The samples of pectoral muscles within 30 min after slaughter were collected to determine meat quality. For Liancheng White ducks and Mianyang Partridge ducks, no pectoralis muscle samples were collected at 42 days of age, and all meat quality indicators at 42 days of age were not determined.

Meat color was evaluated at 45 min post-slaughter using the mid-portion of the right pectoral muscle. L*, a*, and b* values were measured using a portable colorimeter (Miniscan EZ, Hunterlab, Reston, VA, USA).

For shear force determination, muscle samples from the same right pectoral region were vacuum-sealed, submerged in a water bath until the core temperature reached 70 °C, then cooled, and trimmed into 2 cm × 1 cm × 1 cm tendon-free strips. Shear force was measured using a texture analyzer (TMS-PRO, Food Technologies Corporation, Sterling, VA, USA) with the blade aligned parallel to the muscle fiber orientation. The unit of shear force is Newton (symbol: N).

The pH measurements were performed at 45 min and 24 h post-slaughter using a digital pH meter (FEZO PLUS, Mettler Toledo Instruments Shanghai Co., Ltd., Shanghai, China). Muscle samples excised from the right pectoral region were minced into small pieces and homogenized before analysis. The pH meter calibration procedure: preheat the instrument for 15 to 30 min after powering on; rinse the electrode with pure water, then blot it dry with filter paper; use calibration solutions with pH 4.01, 7.00, and 9.21 for linear calibration; after calibration, rinse the electrode again and save the results.

For the drip loss measurements, the middle part of the left side of the pectoral muscle was collected, and a cylindrical shape with a thickness of 1 cm and a diameter of 3 cm was cut in the direction of the muscle fibers. After weighing (W1), the samples were placed in a drip loss tube and refrigerated at 4 °C for 48 h. The surface juice of the meat sample was gently wiped with clean filter paper, and the sample was weighed again (W2). Drip loss was calculated as % = (W2 − W1)/W1 × 100.

### 2.4. Muscle Fiber Characteristics

Muscle fiber characteristics were analyzed in the same duck samples subjected to the aforementioned meat quality measurements, muscle samples excised from the left pectoral region. Muscle samples were carefully excised along the longitudinal axis of muscle fibers and immediately immersed in 4% paraformaldehyde solution at 4 °C for 24 h. After fixation, tissues were dehydrated through a graded ethanol series (70–100%), cleared in xylene, and embedded in paraffin. Serial sections (4 μm thick) were prepared using a Leica RM2235 microtome (RM2235, Leica Biosystems, Nussloch, Germany), mounted on glass slides, and baked at 60 °C for 1 h. Sections were stained with hematoxylin for 5 min, differentiated with 1% hydrochloric acid in ethanol for 5 s, blued with 0.6% ammonia water, and counterstained with eosin for 2 min. Stained slides were dehydrated, cleared in xylene, and coverslipped with neutral balsam. Images were captured using a Nikon Upright Optical microscope (Eclipse Ni-U, Nikon Corporation, Tokyo, Japan) at 200× magnification. Muscle fiber cross—sectional area, nuclear density and diameter were analyzed using Image Pro Plus 6.0 software. Each sample was analyzed in triplicate and at least three random fields of view per section were evaluated.

### 2.5. Muscle Nutritional Components

Muscle nutritional components were analyzed in the same duck samples subjected to the aforementioned meat quality measurements. All remaining meat samples from duck pectoral muscles were stored in a −20 °C freezer. The moisture, fat, and protein content of breast muscle were determined using a fast meat composition analyzer (FoodScan Lab, Hilleroed, Denmark) according to the standard issued “Livestock and poultry meat quality testing: Determination of moisture, protein, and fat—Near-infrared spectroscopy method” [13].

### 2.6. Statistical Analysis

Statistical analysis was performed using IBM SPSS Statistics V22.0. Results are presented as means ± pooled standard error of the mean (SEM). The data were analyzed using one-way analysis of variance followed by Duncan’s multiple-range test.

Principal component analysis (PCA) was implemented using the Factor Analysis module in IBM SPSS Statistics 22.0. The mathematical formula for PCA is expressed as follows:

FACP *=* a_1i_ZX_1_ + a_2i_ZX_2_ + … + a_Pi_ZX_P_

where a_1i_, a_2i_,…, a_Pi_ (i = 1, …, m) represent the eigenvectors corresponding to the eigenvalues of the covariance matrix ∑ of the original variables X, and ZX_1_, ZX_2_, …, ZX_P_ denote their standardized values.

## 3. Results

### 3.1. Growth Performance

The growth performances of the duck breeds are shown in Table 2. According to the results, all ducks gradually gain weight with age, but the weight gain slowing down after 63 days. Body weights were significantly different among the breeds at the same age (*p* < 0.05), The weight was sorted in descending order as follows: C > W > M > L.

### 3.2. Slaughter Performance

The slaughter performances of the duck breeds are presented in Table 3. At 42 days of age, significant differences (*p* < 0.05) were observed in the live weights between the breeds, except between breeds L and M. Dressing percentages were significantly higher and eviscerating percentages were significantly lower in breeds L and M than in the other breeds (*p* < 0.05). The pectoral muscle rates were significantly lower and the leg muscle rates were significantly higher in breeds L and M than in the other breeds (*p* < 0.05). The sebum rate of breed L was significantly higher than the sebum rate of breed M (*p* < 0.05).

At 63 days of age, the live weights were significantly different between the breeds (*p* < 0.05). The eviscerating percentage of breed C was significantly higher than the eviscerating percentages of the other breeds (*p* < 0.05). The pectoral muscle rate of breed W was significantly higher than the pectoral muscle rates of the other breeds (*p* < 0.05). The leg muscle rate of breed L was significantly higher than the leg muscle rates of the other breeds (*p* < 0.05).

At 90 days of age, the live weights were significantly different among the duck breeds (*p* < 0.05), except between breeds L and M. The live weight of breed C was significantly higher than the live weights of the other breeds (*p* < 0.05). The dressing percentage of breed L was significantly lower than the dressing percentages of the other breeds (*p* < 0.05). The eviscerating percentage of breed C was significantly higher than the eviscerating percentages of breeds L and M (*p* < 0.05). The pectoral muscle rates of breeds C and W were significantly higher than the pectoral muscle rates of the other breeds (*p* < 0.05). The leg muscle percentage of breed C was significantly higher than the leg muscle percentages of the other breeds (*p* < 0.05). The sebum rates of breeds L and M were significantly lower than the sebum rates of the other breeds (*p* < 0.05).

A comparison of the same breed across different ages reveals that the pectoral muscle rate tends to increase with age, whereas the leg muscle rate shows the opposite trend; however, the total meat production gradually rises.

### 3.3. Shear Forces

**Figure 1:**
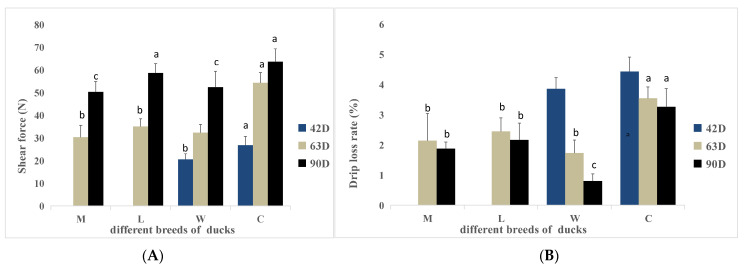
Shear forces and drip loss rate of ducks of different breeds at various ages. (**A**) Shear forces. (**B**) Drip loss rate. a,b,c Values within the same color’s column chart with different superscripts differ significantly at *p* < 0.05.

The shear forces for the duck breeds are shown in Figure 1A. At 42 days of age, the shear force of breed W was significantly lower than the shear force of breed C (*p* < 0.05). At 63 days of age, the shear force of breed C was significantly higher than the shear forces of the other breeds (*p* < 0.05), but there were no significant differences among the other breeds (*p* > 0.05). At 90 days of age, the shear force of breed W was significantly lower than the shear force of breed C (*p* < 0.05), and the shear force of breed M was significantly lower than the shear force of breed C (*p* < 0.05). No significant differences in shear forces were detected between the other breeds (*p* > 0.05). As age increased, shear force increased continuously. The shear force of breed C was significantly higher than the shear forces of the other breeds throughout the growth stages.

### 3.4. Drip Loss Rate

Drip loss rates are shown in Figure 1B. At 42 days of age, no significant differences in the drip loss rates were detected between breeds W and C (*p* > 0.05). At 63 days of age, the drip loss rate of breed C was significantly higher than the drip loss rates of the other breeds (*p* < 0.05). At 90 days of age, the drip loss rates were significantly different between breeds, except between breeds L and M (*p* < 0.05); breed W had the lowest drip loss rate. The drip loss rates tended to decrease as the age increased.

### 3.5. Muscle Fiber Characteristics

**Figure 2:**
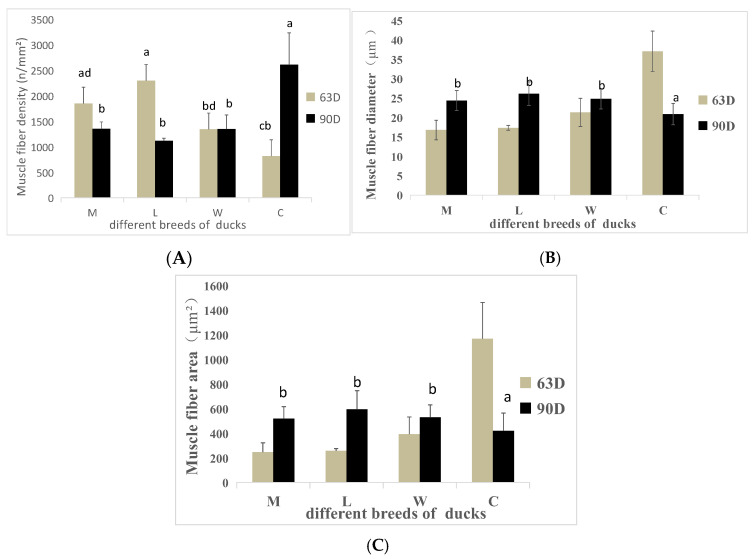
Muscle fiber indicators of ducks of different breeds at various ages. (**A**) Muscle fiber density. (**B**) Muscle fiber diameter. (**C**) Muscle fiber area. a,b,c,d Values within the same color’s column chart with different superscripts differ significantly at *p* < 0.05.

**Figure 3:**
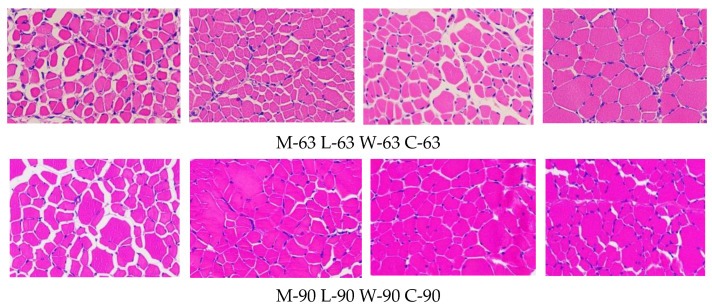
Tissue section of pectoral muscle (200×).

Muscle fiber characteristics are shown in Figure 2 and Figure 3. At 63 days of age, the muscle fiber densities of breeds C and W were significantly lower than the muscle fiber density of breed L (*p* < 0.05). The muscle fiber diameter and area of breed C were significantly higher than the muscle fiber diameters and areas of the other breeds (*p* < 0.05), but no significant differences were detected among the other breeds (*p* > 0.05). At 90 days of age, the muscle fiber density of breed C was significantly higher than the muscle fiber density of breed W (*p* < 0.05) but was not significantly different from the other breeds (*p* > 0.05).

When comparing different ages of the same breed, M, L, and W show a consistent changing trend, while C shows an opposite trend. Muscle fiber densities of breed C tends to increase with age, whereas the muscle fiber diameter and area tends to decrease.

### 3.6. Comparison of pH Values

As shown in Table 4, the 24-h pH of breed W was significantly higher than the pH value of breed C at 42 days of age (*p* < 0.05). At 63 days of age, the 24-h pH values of breeds C and W were significantly lower than the pH values of breeds L and M. At 63 days of age, the 45-min pH of breed W was significantly lower than the 45-min pH of breed L (*p* < 0.05). At 90 days of age, the 45-min pH value of breed W was significantly higher than the 45-min pH value of breed M (*p* < 0.05). For the same breed, the pH at 42 days was higher than the pH at other ages.

### 3.7. Meat Color

The meat color parameters are shown in Table 5. At 42 days of age, the meat color L* of breed W was significantly higher than the L* of breed C (*p* < 0.05). At 63 days of age, the meat color L* of breed L was significantly higher than the L* of the other breeds, but the a* of breed L was significantly lower than the a* of breed W (*p* < 0.05). At 90 days of age, the a* of breed W was significantly lower than the a* of breed M but significantly higher than the a* of breed C (*p* < 0.05). The b* of breed W was significantly lower than the b* of breeds L and M (*p* < 0.05). For the same breed, meat color values L* tended to decrease as the age increased.

### 3.8. Meat Chemical Analysis

**Figure 4:**
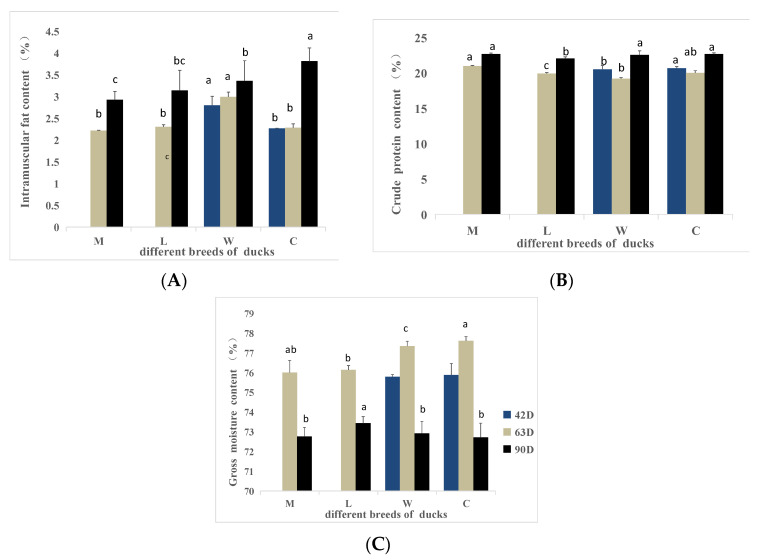
Chemical indicators of different breeds of ducks at different ages. (**A**) The intramuscular fat content. (**B**) The crude protein content. (**C**) The gross moisture content. a,b,c Values within the same color’s column chart with different superscripts differ significantly at *p* < 0.05.

Intramuscular fat contents are shown in Figure 4. The intramuscular fat of breed W was significantly higher than the intramuscular fat of breed C at 42 days of age (*p* < 0.05). At 63 days of age, the intramuscular fat of W was significantly higher than the intramuscular fat of the other breeds (*p* < 0.05). At 90 days of age, the intramuscular fat of breed W was significantly higher than the intramuscular fat of M but significantly lower than the intramuscular fat of C (*p* < 0.05), with no difference compared with breed L (*p* > 0.05). The intramuscular fat increased with age within each breed.

At 42 days of age, the crude protein content of W was significantly lower than the crude protein content of C (*p* < 0.05). At 63 days of age, the crude protein content of L was significantly lower than the crude protein contents of the other breeds (*p* < 0.05). The crude protein content of M was significantly higher than the crude protein of W (*p* < 0.05). At 90 days of age, the crude protein content of L was significantly lower than the crude protein contents of the other breeds (*p* < 0.05). Among all breeds, the crude protein content showed an increasing trend with age after 63 days of age.

No significant differences in the gross moisture content between the W and C breeds were detected at 42 days of age (*p* > 0.05). At 63 days of age, the gross moisture content of C was significantly higher than the gross moisture contents of L and W breeds (*p* < 0.05). At 90 days of age, the gross moisture content of L was significantly higher than the gross moisture contents of the other breeds (*p* < 0.05). Overall, the gross moisture content decreased with age in all breeds after 63 days of age.

### 3.9. Comprehensive Analysis and Evaluation Model

A correlation analysis was performed on breast muscle meat quality results from 42-, 63-, and 90-day-old ducks. As shown in Table 6, eight pairs with significant positive correlations (*p* < 0.01) and ten pairs with significant negative correlations (*p* < 0.01) were identified. Significant positive correlations were detected between 45-min and 24-h pH values and meat color L* and the drip loss rate (*p* < 0.01). Significant negative correlations were detected between meat color a*, meat color b*, and the shear force (*p* < 0.01). Significant positive correlations were detected between meat color L* and the drip loss rate (*p* < 0.01), and significant positive correlations were detected between meat color a* and the shear force (*p* < 0.01). Meat color a* positively correlated with meat color b* and the shear force (*p* < 0.01) and negatively correlated with the drip loss rate (*p* < 0.01). Significant negative correlations were detected between the drip loss rate and shear force (*p* < 0.01). Overall, a high degree of overlapping correlations was detected among these indicators. Thus, these parameters were suitable for PCA to simplify the evaluation of meat quality.

PCA revealed seven indicators of meat quality, including 45-min and 24-h pH, meat color (L*, a, and b*), drip loss, and shear force. The eigenvalue was greater than 0.8 and the cumulative contribution rate reached over 85%. The Kaiser–Meyer–Olkin value (0.713) was >0.5 and the sphericity test was *p* < 0.01, suggesting that the data were suitable for PCA.

After extracting the principal components, as shown in Table 7, the information extracted by all variables exceeded 80%. The first four principal components were extracted and their cumulative contribution rate reached 89.599% (Table 8). Based on the PCA (Table 9 and Table 10), four principal component factor analysis models (Z1/Z2/Z3/Z4) and one comprehensive evaluation model (K) were established. Using this comprehensive evaluation model, the meat quality of the duck breeds ranked in descending order was as follows: W63 > M90 > C63 > L90 > M63 > C90 > L63 > W90 > C42 > W42 (Table 11). Based on this ranking, the meat quality of C ducks was superior to the meat quality of W ducks at 42 days of age. At 63 days of age, the meat qualities of W and C ducks were better than the meat qualities of M and L ducks. At 90 days of age, the meat qualities of M and L ducks were better than the meat qualities of C and W ducks. For the C and W duck breeds, the meat quality at 63 days was better than the meat quality at 90 days and the meat quality at 90 days was better than the meat quality at 42 days. For the M and L duck breeds, the meat quality at 90 days was better than the meat quality at 63 days.

## 4. Discussion

Duck meat boasts a well-balanced nutritional profile, serving as an abundant source of protein, vitamins, and minerals, thus ranking among high-quality animal-derived foods. China is home to over 30 indigenous duck breeds, with distinct meat quality traits across different breeds or strains. Each breed also has an optimal marketing age: fast-growing ducks are marketed at 35–42 days, reaching a market weight of 2.5–3.0 kg; medium-growing hybrid breeds are marketed at 50–70 days, with a market weight of 1.5–2.5 kg; and slow-growing ducks are marketed at 90–120 days, weighing 1.0–1.5 kg at market [14,15].

Breed selection and slaughter age are critical determinants of duck meat quality. Analyzing growth performance, carcass traits, and pectoral muscle quality across different duck breeds and ages can not only lay a theoretical foundation for meat quality evaluation but also yield insights into breed selection and age-specific quality optimization.

### 4.1. Analysis of Growth and Development Patterns in Different Duck Breeds

The growth rates of the four duck breeds, ranked from high to low, are C > W > M > L. At 42 days, the average weight of C reached 2496 g, with a full net carcass rate of 71.72%, and the breast and leg muscle rates reached 23.05%, consistent with previous reports [16,17]. At this stage, the meat production indicators of the C breed met the market requirements. However, the other three breeds did not reach the weight standard at 42 days, especially the M and L duck breeds, which had very low meat production rates. The breast muscle rates of L and M were less than 5% due to the small amount of breast muscle. Thus, the meat quality at 42 days could not be accurately measured. At 63 days, the average market weight of the W breed was 1759 g, with a full net carcass rate of 72.44% and breast and leg muscle rates of 28.95%. All indicators for W met the market requirements at 63 days of age, aligning with the observations of Bai et al. [18]. Nevertheless, the breeding data are inconsistent with the report by Yang [7]. This may be caused by different breeding environments. At 90 days of age, the market weights of L and M reached approximately 1100 g; the full net carcass rate was over 65%, and the breast and leg muscle rates reached over 20%. All the indicators of L and M met the market requirement at 90 days. These four breeds cover the three types of meat ducks demanded by the market. Each breed has specific performance characteristics and meets the processing and production consumer requirements for each meat duck type.

### 4.2. Analysis of Meat Quality in Different Duck Breeds at Various Growth Stages

Meat quality includes taste, aroma, texture, and color [19]. The commonly used meat quality evaluation indicators include physical indicators (meat color, pH, shear force, water-holding capacity, muscle fiber density, and diameter), chemical indicators (moisture, fat, and protein), and flavor substances (fatty acids and amino acids) [20,21].

Tenderness, one of the main edible qualities of meat, refers to the softness and juiciness of meat when chewed. Tenderness can be assessed by measuring shear force; the lower the shear force, the better the tenderness. In this study, the shear force of different duck breeds increased with age, indicating decreased tenderness. In this study, the shear force of different duck breeds increased with age, indicating a decrease in tenderness, which is consistent with the observations of He et al. [22]. When compared at the same time point, the shear force of the W and M breeds was lower than that of the other breeds, while that of the C breed was the highest. Meat tenderness is determined by protein denaturation, intramuscular fat, connective tissue, and myofibrillar structure [3,23]. Smaller muscle fiber diameter, higher muscle fiber density, and higher fat content correlate with more tender and juicy meat. In this experiment, the muscle fiber diameter of the three duck breeds (W, L, and M) increased and the muscle fiber density decreased with age, causing the deterioration of muscle tenderness with age. Thus, morphology and muscle fiber structure play a major role in tenderness [24]. However, in the C duck breed, muscle fiber diameters decreased, muscle fiber density increased, and shear force gradually increased with age. Thus, the tenderness of C may be closely related to muscle connective tissue, the content and cross-linking of collagen in skeletal muscle affect meat tenderness [25]. The specific reasons remain unclear. Due to the limited number of indices measured in this experiment, it is impossible to fully explain this result.

Meat color is an important indicator of quality. The color of pectoral muscles is influenced by breed, diet composition, age, fat content, and pigment level [26]. The color of fresh meat is primarily determined by the relative concentrations of three myoglobin derivatives: the desirable bright red oxymyoglobin, the purple-red myoglobin, and the undesirable brown metmyoglobin [27,28]. The L* value of the meat color parameter represents brightness: the larger the value, the brighter the meat. In this experiment, the muscle L* values decreased and the brightness of the muscle decreased with increased age. The a* value represents redness and is closely related to myoglobin content in the muscle. The a* values of C and W peaked at 63 days of age and the a* values of L and M peaked at 90 days. The b* value represents yellowness and the b* value of all breeds was the highest at 63 days of age and decreased at 90 days of age. The correlation analysis revealed a highly significant negative correlation between L* and a*, a highly significant positive correlation between a* and b*, and no significant correlation between L* and b*. Other studies have drawn conclusions inconsistent with ours [29].

The pH is an important indicator of muscle acidity. After slaughter, the oxidation of muscle glycogen to lactic acid and the decomposition of ATP to produce phosphate decrease the pH in the muscle. Thus, the pH can be used to analyze the rate and intensity of muscle glycogenolysis and is also an important indicator of abnormal meat. The meat pH and the rate at which the pH decreases are influenced by pre- and post-mortem biochemical events involving structural components in muscle cells and the associated connective tissue [30,31]. In this study, the pH of fresh meat from the four duck breeds was 5.7–6.3 within 1 h after slaughter (normal range: 6.0–7.4) and 5.5–6.2 24 h after slaughter (normal range: 5.5–6.0). Thus, the pH values were largely within the normal range of meat pH values.

Muscle fat is an important flavor precursor substance that improves the tenderness, juiciness, and color of meat, making the meat more palatable [32,33,34]. In this study, the fat content in the pectoral muscles of all breeds increases with age, aligning with the observations of Yang et al. [7]. In the comparison of the same age, the muscle fat content was higher in the W breed than in the other breeds. Therefore, the W breed exhibits better meat flavor.

### 4.3. Comprehensive Evaluation of Meat Quality in Different Duck Breeds

The correlation analysis revealed that the drip loss rate positively correlated with pH and meat color, but the shear force significantly correlated with pH, meat color, and the drip loss rate. According to research, the larger the weight, the greater the shear force and drip loss rate and the higher the fat content [35]. Our research results are consistent with this report. PCA reduces multiple variables to a few comprehensive variables, reflecting the information of the original multiple variables. In principal component evaluation, several comprehensive variables can be used to obtain a total ranking. PCA and evaluation are widely used to analyze the production performance and germplasm characteristics of livestock and poultry [36,37,38]. The meat quality of W and C peaked at 63 days of age and the meat quality of M and L peaked at 90 days of age. However, the meat quality and production performance, consumer demand, and feeding costs should all be considered when determining the appropriate marketable age for a breed. Based on our results, the meat production performance of C met market demands at 42 days of age, consistent with previous reports [39]. Longer feeding periods increase input costs, affecting economic benefits. For the other three breeds, the optimal age for meat production and meat quality were consistent. If these breeds are marketed earlier, the meat quality and production will not meet market demands.

## 5. Conclusions

This study systematically compares the growth performance, carcass traits, and meat quality attributes of four duck breeds—Cherry Valley ducks (C), Wuqin 10 meat ducks (W), Liancheng White ducks (L), and Mianyang Partridge ducks (M)—across different growth stages. The results indicate that at all three growth stages, Cherry Valley ducks exhibited higher body weight and meat yield than the other breeds. At 42 days of age, they reached a market weight of 2496 g, with an evisceration rate of 71.72% and a lean meat percentage of 23.05%. Wuqin 10 meat ducks showed excellent meat quality characteristics alongside appropriate meat production. At 63 days of age, they achieved a market weight of 1759 g, with an evisceration rate of 72.44% and a lean meat percentage of 28.95%. Liancheng White ducks and Mianyang Partridge ducks had relatively lower body weight and meat production compared to the other breeds, yet their shear force and water-holding capacity were superior to those of Cherry Valley ducks. By 90 days of age, both breeds reached approximately 1100 g in market weight, with evisceration rates exceeding 65% and lean meat percentages over 20%. At marketable age, meat quality varied among breeds, with the descending ranking as follows: 63-day-old Wuqin 10 ducks > 90-day-old Mianyang Partridge ducks > 90-day-old Liancheng White ducks > 42-day-old Cherry Valley ducks. These findings provide a theoretical reference for evaluating the meat quality of different duck breeds.

## Figures and Tables

**Table 1:** Composition and nutrient level of experimental animal diets.

Item	0–21 D	22–42 D	43–90 D
Ingredients (%)			
Corn	55.25	55.80	62.07
Soybean meal (44%)	28.00	20.00	13.50
Wheat bran	4.00	7.00	6.50
Fish meal	1.50	0.00	0.00
Canola meal	0.00	4.00	5.00
Rice bran	6.00	8.00	9.00
Soybean oil	1.00	1.00	0.00
Limestone	1.20	1.20	1.35
Dicalcium phosphate	1.40	1.40	1.05
Salt	0.35	0.35	0.35
DL-methionine (99%)	0.20	0.18	0.14
L-Lysine (70%)	0.10	0.07	0.04
Premix ^1^	1.00	1.00	1.00
Total	100.00	100.00	100.00
Nutrient levels ^2^ (%)			
ME/(MJ/kg)	12.07	11.80	11.64
CP	19.20	16.99	14.99
Ca	0.90	0.84	0.81
AP	0.42	0.38	0.32
Met	0.49	0.43	0.37
Lys	1.08	0.86	0.70
Met + Cys	0.78	0.71	0.63

^1^ Each kilogram of diet provides vitamin A 9000 IU, vitamin D3 2500 IU, vitamin E 30 IU, vitamin K3 2.5 mg, vitamin B1 2 mg, vitamin B2 8 mg, vitamin B12 0.02 mg, calcium pantothenate 20 mg, niacin 50 mg, folic acid 2 mg, biotin 0.1 mg, iron 80 mg, copper 10 mg, zinc 80 mg, manganese 90 mg, selenium 0.3 mg, and iodine 0.4 mg. ^2^ The nutritional levels are all calculated values. Abbreviations: CP = crude protein; AP = available phosphorus.

**Table 2:** Body weight of ducks of different breeds at various ages.

Age	Gender	C	W	L	M
42 D	Male	2615.73 ± 54.45 ^a^	1316.09 ± 17.48 ^b^	652.80 ± 12.66 ^d^	756.88 ± 13.81 ^c^
	Female	2445.79 ± 34.32 ^a^	1176.71 ± 11.34 ^b^	664.12 ± 5.08 ^d^	752.17 ± 9.84 ^c^
63 D	Male	2845.59 ± 59.15 ^a^	1827.34 ± 7.28 ^b^	972.05 ± 6.60 ^d^	1093.93 ± 16.39 ^c^
	Female	2612.13 ± 50.34 ^a^	1656.81 ± 14.50 ^b^	953.18 ± 5.05 ^d^	1080.02 ± 7.29 ^c^
90 D	Male	3157.28 ± 46.68 ^a^	2066.48 ± 29.51 ^b^	1143.26 ± 4.55 ^d^	1231.80 ± 30.40 ^c^
	Female	2921.85 ± 16.19 ^a^	1929.43 ± 33.50 ^b^	1035.33 ± 14.29 ^d^	1171.26 ± 15.67 ^c^

Data are presented as mean ± SEM. ^a,b,c,d^ Values within a line with different superscripts differ significantly at *p* < 0.05.

**Table 3:** Effect of varieties on slaughter performance in ducks at different ages.

Age	Breeds	LW	DP	EP	PMR	LMR	SR
**42D**	C	2495.67 ± 238.75 ^a^	85.97 ± 1.01 ^b^	71.72 ± 2.27 ^b^	9.09 ± 1.47 ^a^	13.96 ± 1.37 ^b^	25.13 ± 3.76 ^ab^
	W	1240.88 ± 98.31 ^b^	87.48 ± 1.07 ^b^	74.06 ± 0.63 ^a^	6.35 ± 0.82 ^b^	12.86 ± 1.38 ^b^	26.52 ± 2.83 ^ab^
	L	641.00 ± 38.04 ^c^	90.34 ± 0.97 ^a^	66.74 ± 2.48 ^c^	2.08 ± 0.47 ^d^	19.71 ± 1.57 ^a^	28.53 ± 2.58 ^a^
	M	751.50 ± 28.85 ^c^	90.74 ± 3.63 ^a^	66.05 ± 2.46 ^c^	4.33 ± 0.59 ^c^	18.62 ± 1.08 ^a^	23.81 ± 2.36 ^b^
**63D**	C	2665.33 ± 73.40 ^a^	88.66 ± 1.87	74.60 ± 1.10 ^a^	13.78 ± 1.54 ^b^	12.40 ± 1.48 ^c^	24.75 ± 1.97
	W	1758.87 ± 93.58 ^b^	89.11 ± 1.75	72.44 ± 0.79 ^b^	16.96 ± 0.80 ^a^	11.99 ± 1.31 ^c^	24.01 ± 2.64
	L	932.33 ± 50.61 ^d^	89.65 ± 0.91	69.13 ± 1.48 ^c^	9.97 ± 0.45 ^c^	16.87 ± 1.21 ^a^	23.14 ± 3.84
	M	1094.33 ± 26.26 ^c^	88.01 ± 0.41	69.29 ± 0.85 ^c^	11.00 ± 1.49 ^c^	15.31 ± 0.90 ^b^	22.13 ± 2.12
**90D**	C	2987.88 ± 443.86 ^a^	89.04 ± 2.29 ^a^	75.47 ± 7.43 ^a^	15.06 ± 1.53 ^a^	11.73 ± 1.16 ^a^	21.74 ± 5.37 ^a^
	W	1853.33 ± 166.45 ^b^	89.85 ± 0.98 ^a^	70.69 ± 1.85 ^ac^	16.67 ± 2.20 ^a^	6.48 ± 1.47 ^c^	22.52 ± 4.11 ^a^
	L	1043.00 ± 31.92 ^c^	86.93 ± 1.21 ^b^	67.09 ± 1.16 ^b^	12.16 ± 0.86 ^b^	8.38 ± 0.28 ^b^	16.78 ± 2.38 ^b^
	M	1168.50 ± 44.78 ^c^	88.66 ± 0.47 ^a^	69.96 ± 1.78 ^bc^	13.10 ± 0.66 ^b^	7.78 ± 0.41 ^b^	16.87 ± 1.56 ^b^

Data are presented as mean ± SEM. ^a,b,c,d^ Values within a column with different superscripts differ significantly at *p* < 0.05. Abbreviations: LW = live weight; DP = dressing percentage; EP = eviscerating percentage; PMR = pectoral muscle rate; LMR = leg muscle rate; SR = sebum rate.

**Table 4:** Effect of varieties on pH values in ducks at different ages.

Age	Breeds	pH_45min_	pH_24h_
42 D	C	6.20 ± 0.06	6.05 ± 0.03 ^b^
	W	6.29 ± 0.10	6.20 ± 0.08 ^a^
63 D	C	5.86 ± 0.03 ^ab^	5.49 ± 0.02 ^b^
	W	5.84 ± 0.02 ^b^	5.52 ± 0.01 ^b^
	L	6.00 ± 0.12 ^a^	5.62 ± 0.05 ^a^
	M	5.94 ± 0.08 ^ab^	5.61 ± 0.06 ^a^
90 D	C	5.84 ± 0.03 ^ab^	5.89 ± 0.09
	W	5.94 ± 0.10 ^a^	5.78 ± 0.06
	L	5.84 ± 0.09 ^ab^	5.74 ± 0.11
	M	5.82 ± 0.05 ^b^	5.72 ± 0.12

Data are presented as mean ± SEM. ^a,b^ Values within a column with different superscripts differ significantly at *p* < 0.05.

**Table 5:** Effect of varieties on meat color in ducks at different ages.

Age	Breeds	L*	a*	b*
42 D	C	47.89 ± 2.12 ^b^	14.80 ± 1.10	6.61 ± 0.52
	W	51.48 ± 3.47 ^a^	14.75 ± 1.47	7.26 ± 0.17
63 D	C	42.05 ± 1.31 ^c^	19.01 ± 1.17 ^bc^	11.66 ± 1.48
	W	41.74 ± 2.28 ^c^	21.19 ± 0.67 ^a^	10.44 ± 0.96
	L	49.41 ± 2.51 ^a^	18.06 ± 1.48 ^b^	11.57 ± 0.67
	M	45.10 ± 0.51 ^b^	20.13 ± 0.07 ^ac^	11.26 ± 0.85
90 D	C	35.81 ± 1.90 ^a^	17.28 ± 1.51 ^c^	7.93 ± 0.93 ^ab^
	W	33.21 ± 3.06 ^b^	19.91 ± 0.99 ^b^	6.82 ± 0.75 ^b^
	L	36.07 ± 2.46 ^a^	20.95 ± 1.01 ^ab^	8.23 ± 0.68 ^a^
	M	32.27 ± 1.67 ^b^	21.50 ± 1.00 ^a^	8.61 ± 0.64 ^a^

Data are presented as mean ± SEM. ^a,b,c^ Values within a column with different superscripts differ significantly at *p* < 0.05.

**Table 6:** Correlation analysis of different meat quality traits.

Item	pH_45m_	pH_24h_	MCL	MCA	MCB	DLR	SF
pH_45m_	1.00						
pH_24h_	0.80 **	1.000					
MCL	0.54 **	0.34 **	1.00				
MCA	−0.63 **	−0.58 **	−0.68 **	1.00			
MCB	−0.36 **	−0.58 **	0.05	0.43 **	1.00		
DLR	0.38 **	0.34 **	0.42 **	−0.46 **	−0.13	1.00	
SF	−0.49 **	−0.37 **	−0.63 **	0.40 **	0.04	−0.28 **	1.00

** Indicating a highly significant correlation (*p* < 0.01). Abbreviations: MCL = meat color; MCA = meat color a*; MCB = meat color b*; DLR = drip loss rate; SF = shear force.

**Table 7:** Communalities.

Item	Initial	Extraction
pH_45m_	1.000	0.857
pH_24h_	1.000	0.913
MCL	1.000	0.917
MCA	1.000	0.931
MCB	1.000	0.879
DLR	1.000	0.966
SF	1.000	0.809

**Table 8:** Total variance explained.

Component	1	2	3	4
Eigenvalues	3.680	1.327	0.765	0.500
Variance(%)	52.577	18.960	10.922	7.140
Cumulative (%)	52.577	71.537	82.460	89.599

**Table 9:** Matrix and eigenvectors.

Item/Component	1		2		3		4	
	Matrix	Eigenvector	Matrix	Eigenvector	Matrix	Eigenvector	Matrix	Eigenvector
pH_45m_	0.87	0.45	−0.11	−0.09	−0.17	−0.19	0.26	0.36
pH_24h_	0.81	0.42	−0.41	−0.35	−0.14	−0.16	0.26	0.37
MCL	0.75	0.39	0.53	0.46	−0.04	−0.04	−0.27	−0.38
MCA	−0.85	−0.44	0.04	0.03	−0.17	−0.19	0.42	0.59
MCB	−0.46	−0.24	0.79	0.69	−0.03	−0.03	0.20	0.28
DLR	0.59	0.31	0.23	0.20	0.70	0.80	0.26	0.37
SF	−0.65	−0.34	−0.43	−0.38	0.44	0.50	−0.10	−0.13

**Table 10:** Principal component model and comprehensive ranking index.

Component	Model
Z_1_	0.42 Z_pH24h_ + 0.45 Z_pH45m_ + 0.39Z_MCL_ − 0.44Z_MCA_ − 0.24Z_MCB_ + 0.31Z_DLR_ − 0.34Z_SF_
Z_2_	−0.35 Z_pH24h_ − 0.09 Z_pH45m_ + 0.46Z_MCL_ + 0.03Z_MCA_ + 0.69Z_MCB_ + 0.20Z_DLR_ − 0.38Z_SF_
Z_3_	−0.16 Z_pH24h_ − 0.19 Z_pH45m_ − 0.04Z_MCL_ − 0.19Z_MCA_ − 0.03Z_MCB_ + 0.80Z_DLR_ + 0.50Z_SF_
Z_4_	0.37 Z_pH24h_ + 0.36 Z_pH45m_ − 0.38Z_MCL_ + 0.59Z_MCA_ + 0.28Z_MCB_ + 0.37Z_DLR_ − 0.13Z_SF_
K	(3.680Z_1_ + 1.327Z_2_ + 0.765Z_3_ + 0.500Z_4_)/(3.680 + 1.327 + 0.765 + 0.500)

**Table 11:** Principal component scores and comprehensive scores.

Age	Breeds	Z1	Z2	Z3	Z4	K	Ranking
42	C	45.76	−235.09	7.39	0.34	−21.99	9
42	W	60.57	−317.96	−5.9	4.16	−32.12	10
63	C	−15.85	90.61	15.36	0.25	11.78	3
63	W	−15.11	110.71	−7.47	−0.8	13.58	1
63	M	−5.97	47.8	−6	1.92	6.03	5
63	L	−0.35	12.49	−2.8	−5.39	1.68	7
90	C	−3.65	21.65	9.13	−0.48	3.5	6
90	W	−19.48	30.55	−7.09	−2.24	−5.99	8
90	M	−24.99	128.11	−1.32	5.25	12.7	2
90	L	−16.41	81	1.41	−0.91	7.63	4

## Data Availability

All relevant data are within the manuscript. For raw data, please contact the corresponding author.

## Abbreviations

The following abbreviations are used in this manuscript:

LW	Live weight
DP	Dressing percentage
EP	Eviscerating percentage
PMR	Pectoral muscle rate
LMR	Leg muscle rate
SR	Sebum rate
DE	Muscle fiber density
DI	Muscle fiber diameter
AR	Muscle fiber area
MCL	Meat color L*
MCA	Meat color a*
MCB	Meat color b*
DLR	Drip loss rate
SF	Shear force
AP	Available phosphorus
CP	Crude protein

## References

[1] Hou S., Liu L. (2024). Report on the Development of Waterfowl Industry and Technology in 2023. Chin. J. Anim. Sci..

[2] Hou S., Liu L. (2023). Current situation, future development trend and suggestions of waterfowl industry in 2022. Chin. J. Anim. Sci..

[3] Bakhsh A., Hwang Y.-H., Joo S.-T. (2019). Effect of Slaughter Age on Muscle Fiber Composition, Intramuscular Connective Tissue, and Tenderness of Goat Meat during Post-Mortem Time. Foods.

[4] Bai H., Bao Q., Zhang Y., Song Q., Liu B., Zhong L., Zhang X., Wang Z., Jiang Y., Xu Q. (2020). Research Note: Effects of the rearing method and stocking density on carcass traits and proximate composition of meat in small-sized meat ducks. Poult. Sci..

[5] Clinquart A., Ellies-Oury M.P., Hocquette J.F., Guillier L., Santé-Lhoutellier V., Prache S. (2022). Review: On-farm and processing factors affecting bovine carcass and meat quality. Animal.

[6] Weng K., Huo W., Gu T., Bao Q., Hou L., Zhang Y., Zhang Y., Xu Q., Chen G. (2021). Effects of marketable ages on meat quality through fiber characteristics in the goose. Poult. Sci..

[7] Yang C., Li Y., Liu B., Chen A., Bai H., Jiang Y., Chang G., Chen G., Wang Z. (2025). Comparative analysis of duck meat quality in different breeds and age. Food Chem. X.

[8] Tang H., Zhang H., Liu D., Li S., Wang Z., Yu D., Guo Z., Hou S., Zhou Z. (2023). Changes in physical architecture and lipids compounds in skeletal muscle from Pekin duck and Liancheng white duck. Poult. Sci..

[9] Huo W., Weng K., Gu T., Zhang Y., Zhang Y., Chen G., Xu Q. (2021). Effect of muscle fiber characteristics on meat quality in fast- and slow-growing ducks. Poult. Sci..

[10] Kokoszyński D., Arpášová H., Hrnčar C., Żochowska-Kujawska J., Kotowicz M., Sobczak M. (2020). Carcass characteristics, chemical composition, physicochemical properties, texture, and microstructure of meat from spent Pekin ducks. Poult. Sci..

[11] Wang X., Jiang G., Kebreab E., Li J., Feng X., Li C., Zhang X., Huang X., Fang C., Fang R. (2020). HNMR-based metabolomics study of breast meat from Pekin and Linwu duck of different ages and relation to meat quality. Food Res. Int..

[12] Gao Y., Lu J., Zou J., Tang X., Jia X., Fan Y., Ge Q., Chen K., Li H., Liu Y. (2020). Terminology and Measurement Calculation Method for Poultry Performance: NY/T 823-2020.

[13] Qiao X., Bai Y., Wang Y., Xie P., Zhou H., Zou X., Zhang D., Li J., Gao S., Zang M. (2022). Livestock and Poultry Meat Quality Testing: Determination of Moisture, Protein, and Fat—Near-Infrared Spectroscopy Method: GB/T 41366-2022.

[14] Lin R., Li J., Yang Y., Yang Y., Chen J., Zhao F., Xiao T. (2022). Genome-Wide Population Structure Analysis and Genetic Diversity Detection of Four Chinese Indigenous Duck Breeds from Fujian Province. Animals.

[15] Zhang L., Li L., Xin Q., Zhu Z., Miao Z., Zheng N. (2023). Metabolomic characterization of Liancheng white and Cherry Valley duck breast meat and their relation to meat quality. Poult. Sci..

[16] Lv G., Zeng Q., Ding X., Bai S., Zhang K. (2022). Effects of age and diet forms on growth-development patterns, serum metabolism indicators, and parameters of body fat deposition in Cherry Valley ducks. Anim. Biosci..

[17] Hu X., Wang W., Liu L., Wang C., Feng W., Luo Q., Han R., Wang X. (2019). Effects of fat type and emulsifier in feed on growth performance, slaughter traits, and lipid metabolism of Cherry Valley ducks. Poult. Sci..

[18] Bai H., Yang B., Dong Z., Li X., Song Q., Jiang Y., Chang G., Chen G. (2022). Research Note: Effects of cage and floor rearing systems on growth performance, carcass traits, and meat quality in small-sized meat duck. Poult. Sci..

[19] Erasmus S.W., Muller M., Hoffman L.C. (2017). Authentic sheep meat in the European Union: Factors influencing and validating its unique meat quality. J. Sci. Food Agric..

[20] Shi Y., Tu W., Cao M., Sun L., Zhang S., Xu J., He M., Wu C., Zhang D., Dai J. (2024). Comparison of Nutritional Flavor Substances in Meat Between Shanghai Local Pig Breeds and Commercial DLY Breed. Foods.

[21] Mir N.A., Rafiq A., Kumar F.S., Singh V., Shukla V. (2017). Determinants of broiler chicken meat quality and factors affecting them: A review. Int. J. Food Sci. Tech..

[22] He J., Zheng H., Pan D., Liu T., Sun Y., Cao J., Wu Z., Zeng X. (2018). Effects of aging on fat deposition and meat quality in Sheldrake duck. Poult. Sci..

[23] Warner R.D., Wheeler T.L., Ha M., Li X., Bekhit A.E.-D., Morton J., Vaskoska R., Dunshea F.R., Liu R., Purslow P. (2022). Meat tenderness: Advances in biology, biochemistry, molecular mechanisms and new technologies. Meat Sci..

[24] Picard B., Gagaoua M. (2020). Muscle Fiber Properties in Cattle and Their Relationships with Meat Qualities: An Overview. J. Agric. Food Chem..

[25] Wojtysiak D. (2013). Effect of Age on Structural Properties of Intramuscular Connective Tissue, Muscle Fibre, Collagen Content and Meat Tenderness in Pig longissimus lumborum muscle. Folia Biol..

[26] Fletcher D.L., Qiao M., Smith D.P. (2000). The relationship of raw broiler breast meat color and pH to cooked meat color and pH. Poult. Sci..

[27] Orkusz A., Haraf G., Okruszek A., Werenska-Sudnik M. (2017). Lipid oxidation and color changes of goose meat stored under vacuum and modified atmosphere conditions. Poult. Sci..

[28] Ruedt C., Gibis M., Weiss J. (2023). Meat color and iridescence: Origin, analysis, and approaches to modulation. Compr. Rev..

[29] Petracci M., Betti M., Bianchi M., Cavani C. (2004). Color variation and characterization of broiler breast meat during processing in Italy. Poult. Sci..

[30] Ruedt C., Gibis M., Weiss J. (2022). A research note: Effect of pH on meat iridescence in precooked cured pork. BMC Res. Notes.

[31] Hughes J.M., Oiseth S.K., Purslow P.P., Warner R.D. (2014). A structural approach to understanding the interactions between colour, water-holding capacity and tenderness. Meat Sci..

[32] Fu Y., Cao S., Yang L., Li Z. (2022). Flavor formation based on lipid in meat and meat products: A review. J. Food Biochem..

[33] Khan M.I., Jo C., Tariq M.R. (2015). Meat flavor precursors and factors influencing flavor precursors—A systematic review. Meat Sci..

[34] Li X., Fu X., Yang G., Du M. (2020). Review: Enhancing intramuscular fat development via targeting fibro-adipogenic progenitor cells in meat animals. Animal.

[35] Hiscock H.M., Leishman E.M., Vanderhout R.J., Adams S.M., Mohr J., Wood B.J., Baes C.F., Barbut S. (2022). Describing the relationships among meat quality traits in domestic turkey (*Meleagris gallopavo*) populations. Poult. Sci..

[36] Tan F., Li D., Kaewkot C., Wu H., Świąder K., Yu H., Chen C., Chumngoen W. (2022). Application of principal component analysis with instrumental analysis and sensory evaluation for assessment of chicken breast meat juiciness. Br. Poult. Sci..

[37] Negash F. (2021). Application of principal component analysis for predicting body weight of Ethiopian indigenous chicken populations. Trop. Anim. Health Prod..

[38] Li M., Zhou Z., Zhang Q., Zhang J., Suo Y., Liu J., Shen D., Luo L., Li Y., Li C. (2024). Multivariate analysis for data mining to characterize poultry house environment in winter. Poult. Sci..

[39] Cao Z., Gao W., Zhang Y., Huo W., Weng K., Zhang Y., Li B., Chen G., Xu Q. (2021). Effect of marketable age on proximate composition and nutritional profile of breast meat from Cherry Valley broiler ducks. Poult. Sci..

